# Right-to-left shunt is associated with reduced cerebral vascular reactivity in migraine patients with aura

**DOI:** 10.3389/fneur.2026.1800173

**Published:** 2026-09-08

**Authors:** Changhong Yuan, Yingchun Zhu, Yu Wang, Lu Zhang

**Affiliations:** 1 Department of Neurology, Anhui No. 2 Provincial People's Hospital, Hefei, China; 2 Department of Neurology, The First Affiliated Hospital of Anhui Medical University, Hefei, China; 3 Department of Neurology, Anhui Public Health Clinical Center, Hefei, China

**Keywords:** cerebrovascular reactivity, migraine with aura, patent foramen ovale, right-to-left shunt, transcranial Doppler

## Abstract

**Objective:**

To investigate whether right-to-left shunt (RLS) associated with patent foramen ovale (PFO) is associated with altered cerebrovascular reactivity (CVR) in migraine patients with aura.

**Methods:**

This cross-sectional observational study enrolled migraine patients with aura who attended the outpatient clinic of Anhui No. 2 Provincial People's Hospital between December 2022 and September 2025. All patients underwent contrast-enhanced transcranial Doppler (c-TCD) examination, confirmed by contrast transthoracic echocardiography (c-TTE), to detect RLS and determine PFO status. PFO-negative patients served as the internal control group. RLS was graded (0–3) based on the number of microembolic signals (MES), and patients were further classified as having either latent RLS (microbubbles appearing only after Valsalva maneuver) or persistent RLS (microbubbles appearing at rest). Cerebrovascular reactivity was indirectly assessed by measuring the breath-holding index (BHI) via transcranial Doppler. BHI differences were compared between PFO-positive (+) and PFO-negative (–) groups, between latent and persistent PFO groups, and across different RLS grade groups.

**Results:**

Compared with the PFO (–) group, the PFO (+) group exhibited significantly reduced BHI values in the bilateral middle cerebral arteries (MCAs) and bilateral posterior cerebral arteries (PCAs; all *P* < 0.05). The reduction in BHI became more pronounced with increasing RLS severity grade, showing a significant monotonic trend (Spearman's ρ ranging from −0.401 to −0.466, all *P* < 0.001). Furthermore, migraine patients with aura who had persistent PFO demonstrated a greater decrease in BHI than those with latent PFO compared with the PFO (–) group (*P* < 0.05).

**Conclusion:**

RLS associated with PFO is associated with reduced CVR in migraine patients with aura, with more pronounced reductions in cases of large shunts and persistent shunt types.

## 1 Introduction

Migraine is a prevalent neurological disorder with a high incidence rate, significantly diminishing patients' quality of life. Numerous observational studies and systematic reviews have confirmed that migraine, particularly migraine with aura, increases the risk of cerebrovascular events, especially ischemic stroke (1, 2). Among migraine patients, females with aura are more susceptible to ischemic stroke and may develop migraine-related infarction, which predominantly occurs in the posterior circulation of young women (3). Patent foramen ovale (PFO) is notably more common in migraine patients with aura compared with healthy controls, and closure of PFO can alleviate migraine symptoms (4, 5). Although PFO is more prevalent in this patient group and may partially explain the occurrence of cryptogenic stroke, it does not account for the increased prevalence of asymptomatic posterior circulation infarction or white matter lesions observed in these patients (6, 7). Migraine patients exhibit impaired anterior circulation vascular function, characterized by higher mean blood flow velocity, elevated pulsatility index (PI, reflecting arterial stiffness), and reduced cerebrovascular reactivity to hypercapnia (CVR, reflecting vascular endothelial relaxation function) (8). Additionally, studies have shown reduced CVR in the posterior cerebral artery of migraine patients. Moreover, earlier age of onset and longer disease duration are associated with a more pronounced reduction in posterior cerebral artery CVR (9). The foramen ovale is a crucial fetal structure that typically closes after birth; however, it remains open in approximately 25% of healthy individuals, a condition known as PFO (10, 11). PFO is the most common right-to-left shunt (RLS), accounting for approximately 95% of all RLS cases (12). Both PFO and cerebral hemodynamic impairment are among the pathophysiological mechanisms proposed to explain the increased stroke risk in migraine patients with aura. Therefore, investigating the relationship between PFO and CVR in these patients is of great significance. This study aimed to investigate whether RLS associated with PFO is associated with altered CVR in migraine patients with aura.

## 2 Methods

### 2.1 Study design and participants

Participants were consecutively recruited from the outpatient clinic of the Department of Neurology, Anhui No. 2 Provincial People's Hospital, between December 2022 and September 2025. Patients who attended the headache or neurology outpatient clinics and met the diagnostic criteria for migraine with aura were enrolled. The diagnosis of migraine with aura was made by specialized neurologists according to the International Classification of Headache Disorders, 3rd edition (ICHD-3, 2018) (13). The specific diagnostic criteria were as follows: (A) at least two attacks fulfilling criteria B and C; (B) at least one of the following fully reversible aura symptoms: visual, sensory, speech and/or language, motor, brainstem, or retinal; (C) at least three of the following six features: (1) at least one aura symptom develops gradually over ≥5 min, (2) two or more symptoms occur in succession, (3) each individual aura symptom lasts 5–60 min, (4) at least one aura symptom is unilateral, (5) at least one aura symptom is positive, (6) the aura is accompanied, or followed within 60 min, by headache. Aura characteristics were verified through structured clinical interviews conducted by physicians at the time of diagnosis. For cases with diagnostic uncertainty, patients were asked to complete standardized headache diaries to prospectively document the characteristics of subsequent attacks. All diagnoses were confirmed by a second senior neurologist.

The inclusion criteria were as follows: (1) age between 18 and 50 years; (2) diagnosis of migraine with aura.

The exclusion criteria were as follows: (1) migraine attack within the past 24 h; (2) presence of risk factors that may impair CVR, including hypertension, diabetes, dyslipidemia, coronary heart disease, stroke, current or previous smoking history, pregnancy, or oral contraceptive use; (3) body mass index (BMI) >30 kg/m^2^; (4) current use of migraine prophylactic medications, such as calcium channel blockers; (5) concurrent severe systemic diseases or genetic disorders; (6) insufficient temporal bone acoustic windows, or presence of >50% major vascular stenosis detected on carotid ultrasound or cranial magnetic resonance angiography (MRA). All participants were required to avoid coffee, tea, and smoking for 24 h prior to transcranial Doppler (TCD) assessment.

This was a cross-sectional observational study, and all participants were migraine patients with aura. Based on contrast-enhanced transcranial Doppler (c-TCD) results, patients were divided into PFO-positive and PFO-negative groups, with the latter serving as the internal control group. The decision to include only migraine patients with aura and not to establish a healthy control group was based on the fact that these patients inherently have intrinsic cerebrovascular dysfunction (e.g., reduced CVR). Including healthy controls would make it difficult to distinguish whether between-group differences were attributable to migraine itself or to PFO. The use of an internal control allowed for a more accurate assessment of the effect of PFO-related RLS on CVR in this patient population. This study was conducted in accordance with the ethical guidelines of the Declaration of Helsinki and was approved by the Ethics Committee of Anhui No. 2 Provincial People's Hospital. All participants provided written informed consent.

### 2.2 Contrast-enhanced transcranial Doppler (c-TCD) detection and assessment of PFO

PFO was assessed using contrast-enhanced transcranial Doppler (c-TCD) examination, confirmed by contrast transthoracic echocardiography (c-TTE). Prior to the c-TCD examination, patients were instructed on how to perform a standardized Valsalva maneuver (VM).

#### 2.2.1 Contrast agent preparation

The patient was placed in a supine position, and a 20-gauge indwelling needle was inserted into the right antecubital vein to withdraw 0.5 mL of autologous blood. A three-way stopcock was used to connect two 10 mL syringes, and 8.5 mL of normal saline, 1 mL of air, and 0.5 mL of autologous blood were mixed and vigorously agitated between the two syringes for 30 cycles to prepare the agitated saline contrast agent.

#### 2.2.2 Injection and monitoring protocol

Each patient underwent three contrast agent injections, with an interval of at least 5 min between injections to ensure complete clearance of microbubbles from the circulation. TCD was used to continuously monitor middle cerebral artery (MCA) blood flow and record the number of microembolic signals (MES). The first injection (resting state) was performed with the patient breathing calmly (without VM) via rapid bolus injection to detect persistent RLS (spontaneous shunt at rest). The second and third injections (Valsalva state) were performed with the contrast agent injected 5 s before the start of VM, after which the patient performed VM for 10 s to detect latent RLS (shunt induced only by elevated intrathoracic pressure).

#### 2.2.3 Outcome assessment and definitions

The maximum number of MES recorded in the MCA at rest and after VM was used as an estimate of the degree of shunt. If RLS occurred at rest, it was classified as persistent RLS; if RLS appeared only after VM, it was classified as latent RLS (14).

#### 2.2.4 RLS grading criteria

According to the Chinese expert recommendations on the management of patent foramen ovale (15), RLS was classified into four grades based on the number of microembolic signals (MES): Grade 0 (negative): no MES detected; Grade 1 (mild): 1 ≤ MES ≤ 10; Grade 2 (moderate): MES >10 without the “rain curtain” phenomenon; Grade 3 (severe): presence of a “rain curtain” or “snowstorm” phenomenon (individual MES could not be distinguished on the spectrum). Total RLS included both persistent and latent RLS (14).

### 2.3 Breath-holding index measurement

Intracranial vascular assessment was performed using an EDAN TDD-IIC transcranial Doppler ultrasound system (EDAN, Shenzhen, China). Cerebrovascular reactivity (CVR) was indirectly assessed by measuring the breath-holding index (BHI). Examinations were conducted in a quiet environment in a dedicated TCD testing room by trained neuro-ultrasonographers who were blinded to the study protocol and group assignments. Participants were instructed to avoid strenuous exercise, smoking, alcohol consumption, and intake of tea and coffee for 24 h prior to the examination. Pre-procedural simulation training was conducted to ensure that patients mastered the breath-holding technique.

#### 2.3.1 Breath-holding test procedure

The patient lay supine on the examination table, and a 2 MHz pulse probe was fixed to the temporal window using a monitoring headrest. The probe angle was adjusted to obtain optimal blood flow signals of the MCA at a depth of 50–60 mm and the posterior cerebral artery (PCA) at a depth of 60–70 mm. Mean blood flow velocities of the bilateral MCA and PCA were recorded at rest. Participants were then instructed to hold their breath for 30 s after a normal expiration, and the mean blood flow velocities of each vessel and the actual breath-holding duration were recorded at the end of breath-holding. The procedure was repeated after a 5-min rest. Each patient completed two independent tests for each vessel (left MCA, right MCA, left PCA, and right PCA).

#### 2.3.2 BHI calculation

BHI = (mean blood flow velocity after breath-holding – mean blood flow velocity at rest) × 100/(mean blood flow velocity at rest × breath-holding duration in s). The final BHI for each vessel was taken as the average of the two test values.

#### 2.3.3 Quality control

(1) All recordings had to meet the following criteria: stable blood flow signal envelope, adequate temporal window penetration, and no significant motion artifact during breath-holding; (2) the interval between the two breath-holding tests was at least 5 min to ensure hemodynamic recovery to baseline; (3) all enrolled patients were required to successfully complete two acceptable breath-holding tests; those who were unable to obtain stable recordings due to inadequate signal quality or technical reasons were excluded.

### 2.4 Statistical analysis

Statistical analyses were performed using SPSS 27.0 (IBM Corp., Armonk, NY, USA), R software (version 4.5.2), and GraphPad Prism 10 (GraphPad Software, San Diego, CA, USA). A *P*-value < 0.05 was considered statistically significant.

The Shapiro–Wilk test was used to assess normality, and Levene's test was used to assess homogeneity of variances. Based on the results of normality and homogeneity tests, descriptive statistics were presented as follows: normally distributed continuous data were expressed as mean ± standard deviation (SD); non-normally distributed data were expressed as median (interquartile range, IQR). Categorical variables were presented as frequencies (percentages).

For two-group comparisons, continuous variables that were normally distributed with homogeneous variances were analyzed using the independent samples *t*-test; those that were not normally distributed or had unequal variances were analyzed using the Mann–Whitney *U*-test. Categorical variables were analyzed using the chi-square test.

For multi-group comparisons (≥3 groups), one-way analysis of variance (ANOVA) was used, followed by Fisher's least significant difference (LSD) test for *post-hoc* pairwise comparisons.

#### 2.4.1 Trend test

To verify the monotonic trend between RLS grade and BHI, RLS grade (0, 1, 2, and 3) was treated as an ordinal variable, and Spearman's rank correlation analysis was used to test for trend, with Spearman's rank correlation coefficient (ρ) and its *P*-value calculated.

#### 2.4.2 Post-hoc power analysis

As this was an exploratory study, no a priori sample size calculation was performed. *Post-hoc* power analysis was used to evaluate the ability of the current sample size to detect the primary findings. Using a two-independent-samples *t*-test framework (two-sided, α = 0.05), Cohen's *d* effect size was calculated based on the mean difference and pooled standard deviation of BHI between the PFO (+) and PFO (–) groups, and power was estimated based on a non-central *t*-distribution. Effect sizes were interpreted according to conventional criteria: *d* = 0.2 was considered a small effect, *d* = 0.5 a medium effect, and *d* = 0.8 a large effect. The 95% confidence intervals for Cohen's *d* reported in the text were estimated using the non-central *t*-distribution approximation method.

## 3 Results

### 3.1 Baseline and clinical characteristics of study participants

A total of 82 migraine patients with aura were initially recruited. After screening according to the exclusion criteria, 5 patients with >50% major cerebral artery stenosis, six patients with cerebrovascular risk factors, four patients using migraine prophylactic medications, three patients with insufficient temporal bone windows, and two patients unable to cooperate with the Valsalva maneuver or breath-holding test were excluded, resulting in 62 patients included in the final analysis.

The 62 patients had a mean age of 35.66 ± 8.51 years, 40 (64.52%) were female, and the median disease duration was 10 years (IQR: 6–12 years). Based on c-TCD results, 41 patients (66.1%) were PFO-positive, including 21 with RLS grade 1, 8 with grade 2, and 12 with grade 3; 20 had latent RLS and 21 had persistent RLS. Twenty-one patients (33.9%) were PFO-negative. There were no significant differences between the PFO-positive and PFO-negative groups in age, sex, BMI, or disease duration (all *P* > 0.05), indicating that the baseline characteristics of the two groups were comparable (Table 1).

**Table 1:** Demographic and clinical characteristics of migraine patients with aura, with and without PFO (*n* = 62). Quantitative data are expressed as mean ± SD, and qualitative variables are expressed as frequency (proportion).

Characteristic	All (*n* = 62)	PFO (–) (*n* = 21)	PFO (+) (*n* = 41)	*t/Z*/*χ^2^*	*P*-value
Age (years)	35.66 ± 8.51	33.95 ± 7.65	36.54 ± 8.88	1.135	0.261
Female (*n*, %)	40 (64.52)	14 (66.67)	26 (63.41)	0.064	0.800
BMI (kg/m^2^)	23.21 ± 3.72	22.86 ± 3.89	23.39 ± 3.66	0.531	0.597
Duration (years, median, IQR)	10 (6, 12)	9 (6, 12)	10 (6, 12)	0.246	0.805

PFO, patent foramen ovale; BMI, body mass index.

### 3.2 Comparison between the PFO (–) and PFO (+) groups

At baseline, there were no significant differences in mean blood flow velocities of the left middle cerebral artery (LMCA), right middle cerebral artery (RMCA), left posterior cerebral artery (LPCA), and right posterior cerebral artery (RPCA) between the PFO (–) and PFO (+) groups (all *P* > 0.05, Table 2). After breath-holding, both mean blood flow velocities and BHI of the four vessels in the PFO (+) group were significantly lower than those in the PFO (–) group (all *P* < 0.001, Table 2).

**Table 2:** Comparison of maximum flow velocity before and after breath-holding, and BHI between the PFO-positive and PFO-negative groups.

Variable	PFO (–) (*n* = 21)	PFO (+) (*n* = 41)	*t*	*P*-value
Baseline				
L MCA-V (cm/s)	61.95 ± 6.26	61.76 ± 5.80	0.132	0.903
R MCA-V (cm/s)	61.43 ± 6.23	61.37 ± 5.98	0.039	0.969
L PCA-V (cm/s)	44.29 ± 6.19	42.80 ± 5.50	0.962	0.340
R PCA-V (cm/s)	44.05 ± 5.55	42.85 ± 5.03	0.855	0.396
After breath-holding				
L MCA-V (cm/s)	88.38 ± 9.09	81.10 ± 6.99	3.498	0.001
R MCA-V (cm/s)	88.19 ± 8.53	80.24 ± 6.79	3.993	< 0.001
L PCA-V (cm/s)	61.57 ± 8.27	55.46 ± 6.36	3.226	0.002
R PCA-V (cm/s)	61.42 ± 7.91	55.20 ± 6.43	3.336	0.001
BHI				
L MCA	1.43 ± 0.32	1.06 ± 0.35	4.088	< 0.001
R MCA	1.46 ± 0.32	1.04 ± 0.33	4.766	< 0.001
L PCA	1.31 ± 0.29	1.00 ± 0.32	3.684	< 0.001
R PCA	1.33 ± 0.33	0.97 ± 0.33	3.975	< 0.001

PFO, patent foramen ovale; MCA, middle cerebral artery; PCA, posterior cerebral artery; BHI, breath-holding index; V, velocity.

The Cohen's *d* effect sizes for BHI differences between the two groups were: LMCA *d* = 1.06 (95% CI: 0.50–1.62, power = 0.92), RMCA *d* = 1.24 (95% CI: 0.66–1.81, power = 0.97), LPCA *d* = 0.96 (95% CI: 0.40–1.52, power = 0.87), and RPCA *d* = 1.03 (95% CI: 0.47–1.59, power = 0.91). All primary comparisons had power exceeding 0.80, indicating that the current sample size was sufficient to detect PFO-status-related BHI differences.

### 3.3 Comparison between the PFO (–) group and RLS grade subgroups

**Figure 1:**
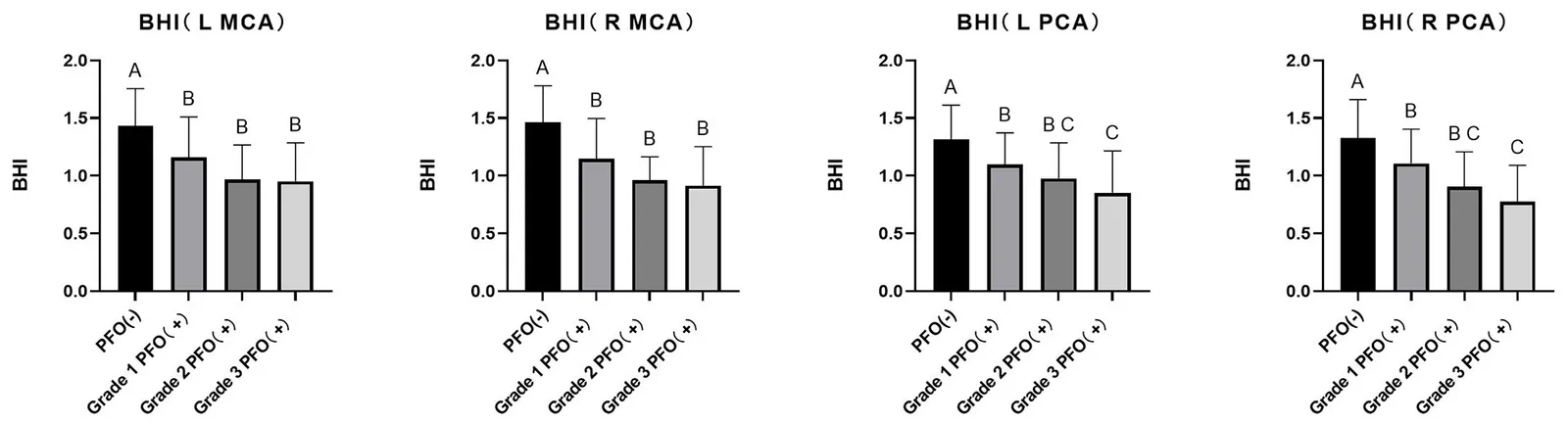
Comparison of BHI among the PFO (–), grade 1 PFO (+), grade 2 PFO (+), and grade 3 PFO (+) groups. Data are presented as mean ± SD, with error bars representing SD. Different letters above the bars indicate statistically significant differences between groups (*P* < 0.05); the same letter indicates no statistically significant difference. RLS, right-to-left shunt; BHI, breath-holding index.

Based on RLS grade, the PFO (+) group was divided into three subgroups: grade 1 (*n* = 21), grade 2 (*n* = 8), and grade 3 (*n* = 12). There were no significant differences in baseline mean blood flow velocities among the groups (all *P* > 0.05). After breath-holding, the differences in blood flow velocities and BHI of the four vessels among the groups were statistically significant (all *P* < 0.05, Table 3). Further pairwise comparisons (Figure 1) showed that for bilateral MCAs, BHI differed significantly between the PFO (–) group and each PFO (+) subgroup (all *P* < 0.05), but no significant differences were observed among the three PFO (+) subgroups (all *P* > 0.05). For bilateral PCAs, there were significant differences between the PFO (–) group and each PFO (+) subgroup (all *P* < 0.01), and the difference between grade 1 and grade 3 groups was also significant (*P* < 0.05), with the grade 3 group showing the most pronounced BHI reduction.

**Table 3:** Comparison of maximum flow velocity before and after breath-holding, and BHI among the PFO-negative group and RLS grade 1, grade 2, and grade 3 subgroups.

Variable	Grade 1 PFO (+) (*n* = 21)	Grade 2 PFO (+) (*n* = 8)	Grade 3 PFO (+) (*n* = 12)	PFO (–) (*n* = 21)	*F*	*P*-value
Baseline L MCA-V(cm/s)	60.95 ± 5.07	62.75 ± 4.62	62.50 ± 7.72	61.95 ± 6.26	0.263	0.852
Baseline R MCA-V (cm/s)	60.57 ± 4.62	62.63 ± 5.71	61.92 ± 8.23	61.43 ± 6.23	0.263	0.852
Baseline L PCA-V (cm/s)	42.48 ± 4.98	43.13 ± 5.14	43.17 ± 6.90	44.29 ± 6.19	0.345	0.793
Baseline R PCA-V (cm/s)	42.52 ± 4.80	42.75 ± 5.12	43.50 ±5.71	44.05 ± 5.55	0.325	0.808
After breath-holding L MCA-V (cm/s)	81.90 ± 7.19	80.75 ± 4.86	79.92 ± 8.14	88.38 ± 9.09	4.146	0.010
After breath-holding R MCA-V (cm/s)	81.14 ± 5.89	80.63 ± 7.80	78.42 ± 7.79	88.19 ± 8.53	5.576	0.002
After breath-holding L PCA-V (cm/s)	56.38 ± 6.47	55.50 ± 5.26	53.83 ± 7.00	61.57 ± 8.27	3.736	0.016
After breath-holding R PCA-V (cm/s)	56.48 ± 5.63	54.25 ± 6.67	53.58 ± 7.63	61.43 ± 7.91	4.173	0.010
BHI (L MCA)	1.16 ± 0.35	0.97 ± 0.29	0.95 ± 0.33	1.43 ± 0.32	6.951	< 0.001
BHI (R MCA)	1.15 ± 0.35	0.96 ± 0.20	0.92 ± 0.34	1.46 ± 0.32	9.409	< 0.001
BHI (L PCA)	1.10 ± 0.36	0.98 ± 0.31	0.85 ± 0.27	1.31 ± 0.30	6.425	0.001
BHI (R PCA)	1.11 ± 0.30	0.91 ± 0.30	0.78 ± 0.32	1.33 ± 0.33	8.845	< 0.001

PFO, patent foramen ovale; RLS, right-to-left shunt; MCA, middle cerebral artery; PCA, posterior cerebral artery; BHI, breath-holding index; V, velocity.

The effect sizes for BHI differences between the RLS grade 3 group and the PFO (–) group were the largest (left PCA: *d* = 1.48, 95% CI: 0.80–2.16, power = 0.87; right PCA: *d* = 1.55, 95% CI: 0.85–2.24, power = 0.91).

To further verify the monotonic trend between RLS grade and BHI, RLS grade (0, 1, 2, and 3) was treated as an ordinal variable, and Spearman's rank correlation analysis was used for trend testing. The results showed that BHI of all four vessels was significantly negatively correlated with RLS grade (left MCA: ρ = −0.401, *P* < 0.001; right MCA: ρ = −0.425, *P* < 0.001; left PCA: ρ = −0.448, *P* < 0.001; right PCA: ρ = −0.466, *P* < 0.001), indicating that higher RLS grade was associated with greater BHI reduction. The negative correlation was more pronounced in the posterior circulation (PCA) than in the anterior circulation (MCA).

### 3.4 Comparison among the PFO (–) group, latent PFO (+) group, and persistent PFO (+) group

Based on the persistence of RLS, the PFO (+) group was further divided into a latent PFO (+) group (*n* = 20) and a persistent PFO (+) group (*n* = 21). At baseline, there were no significant differences in mean blood flow velocities of the four vessels among the three groups (all *P* > 0.05, Table 4). After breath-holding, the differences in blood flow velocities and BHI among the groups were statistically significant (all *P* < 0.05, Table 4).

**Table 4:** Comparison of maximum flow velocity before and after breath-holding, and BHI among the PFO-negative, latent PFO-positive, and persistent PFO-positive groups.

Variable	Latent PFO (+) (*n* = 20)	Persistent PFO (+) (*n* = 21)	PFO (–) (*n* = 21)	*F*	*P*-value
Baseline					
L MCA-V (cm/s)	60.55 ± 5.76	62.90 ± 5.74	61.95 ± 6.26	0.815	0.448
R MCA-V (cm/s)	59.65 ± 5.49	63.00 ± 6.09	61.43 ± 6.23	1.622	0.206
L PCA-V (cm/s)	41.50 ± 4.99	44.05 ± 5.78	44.29 ± 6.19	1.499	0.232
R PCA-V (cm/s)	41.15 ± 4.31	44.48 ± 5.22	44.05 ± 5.55	2.596	0.083
After breath-holding					
L MCA-V (cm/s)	80.95 ± 7.47	81.24 ± 6.71	88.38 ± 9.09	6.024	0.004
R MCA-V (cm/s)	80.05 ± 6.63	80.43 ± 7.10	88.19 ± 8.53	7.854	0.001
L PCA-V (cm/s)	55.20 ± 5.96	55.71 ± 6.86	61.57 ± 8.27	5.148	0.009
R PCA-V (cm/s)	54.70 ± 5.95	55.67 ± 6.99	61.43 ± 7.91	5.586	0.006
BHI					
L MCA	1.14 ± 0.40	0.98 ± 0.27	1.43 ± 0.32	9.713	< 0.001
R MCA	1.16 ± 0.35	0.93 ± 0.29	1.46 ± 0.32	14.62	< 0.001
L PCA	1.11 ± 0.23	0.90 ± 0.37	1.31 ± 0.30	9.726	< 0.001
R PCA	1.10 ± 0.26	0.85 ± 0.35	1.33 ± 0.33	11.924	< 0.001

PFO, patent foramen ovale; MCA, middle cerebral artery; PCA, posterior cerebral artery; BHI, breath-holding index; V, velocity.

**Figure 2:**
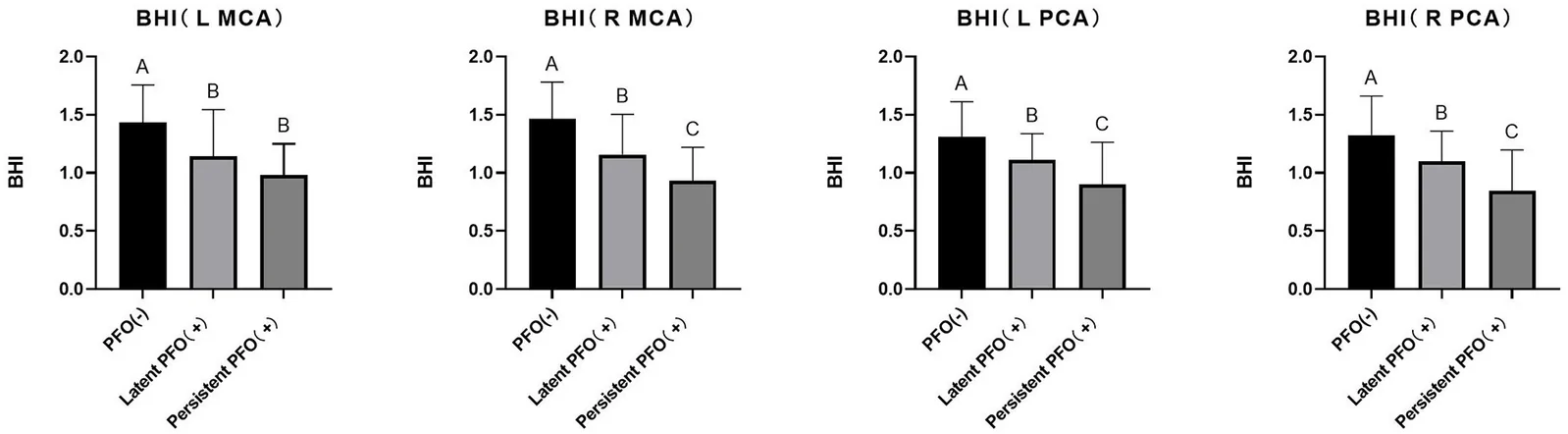
Comparison of BHI among the PFO (–), latent PFO (+), and persistent PFO (+) groups. Data are presented as mean ± SD, with error bars representing SD. Different letters above the bars indicate statistically significant differences between groups (*P* < 0.05); the same letter indicates no statistically significant difference. RLS, right-to-left shunt; BHI, breath-holding index.

Pairwise comparisons (Figure 2) showed that for the left MCA, BHI differed significantly between the PFO (–) group and each PFO (+) subgroup (all *P* < 0.05), but no significant difference was observed between the latent and persistent PFO (+) groups (*P* > 0.05). For the right MCA and bilateral PCAs, pairwise differences among the three groups were all statistically significant (all *P* < 0.05), with the persistent PFO (+) group showing the most pronounced BHI reduction.

The effect sizes for BHI differences between the persistent PFO (+) group and the PFO (–) group (left PCA: *d* = 1.26, 95% CI: 0.64–1.88; right PCA: *d* = 1.30, 95% CI: 0.67–1.92) were larger than those between the latent PFO(+) group and the PFO(–) group (left PCA: *d* = 0.67, 95% CI: 0.09–1.24; right PCA: *d* = 0.64, 95% CI: 0.06–1.21).

## 4 Discussion

This study used PFO-negative migraine patients with aura as internal controls to evaluate the independent effect of PFO on CVR. The main findings were: (1) PFO-positive patients had significantly lower BHI than PFO-negative patients, particularly in the posterior circulation; (2) the degree of BHI reduction showed a dose-dependent relationship with RLS grade and shunt persistence. The balanced baseline characteristics between the two groups (Table 1) ensured the validity of the comparisons.

Our findings are consistent with those of Khasiyev et al. (16), who reported that migraine patients with a large right-to-left shunt (RLS) had significantly lower middle cerebral artery (MCA) breath-holding index (BHI) than those without RLS (1.04 ± 0.67 vs. 1.43 ± 0.39, *p* = 0.032), suggesting that PFO-related RLS is associated with impaired cerebrovascular vasodilatory capacity, particularly in the presence of large shunts. Microemboli in venous blood (e.g., thrombi, air emboli, and fat emboli) and high concentrations of metabolites (e.g., 5-hydroxytryptamine induced by platelet aggregation) can bypass the pulmonary circulation and directly enter the cerebral circulation through RLS. Prolonged exposure to these substances may impair cerebrovascular endothelial function, thereby leading to reduced cerebrovascular reactivity (CVR) (17, 18). Migraine patients inherently exhibit interictal CVR abnormalities, particularly in the posterior cerebral artery (PCA) (9), which is associated with diminished vasodilatory capacity (19). Further studies have confirmed that migraine patients have selective cerebrovascular endothelial dysfunction (especially in the posterior circulation), without significant impairment of systemic endothelial function (20), suggesting that posterior circulation vessels may be more susceptible to RLS-related harmful substances. Reduced CVR, together with endothelial impairment, increases the risk of cerebrovascular events in migraine patients. Even in healthy young and middle-aged migraine patients, reduced CVR is significantly associated with an increased burden of deep white matter demyelinating lesions (21, 22).

Migraine with aura is closely associated with PFO, particularly with persistent and large shunts (14). The present study found that patients with higher RLS grades and persistent shunts had more pronounced BHI reductions. Higher RLS grades are associated with a greater total burden of microemboli entering the cerebral circulation and increased exposure to harmful substances, thereby exacerbating CVR impairment. Lim et al. (23), in a cohort study of 234 patients with cryptogenic stroke and PFO, found that patients with large shunts had a significantly higher risk of recurrent ischemic stroke than those with small shunts, and that large shunt size was an independent predictor of stroke recurrence. Pezzini et al. (24) used CT perfusion imaging to examine the ischemic penumbra in patients with cerebral infarction and found that migraine patients, especially those with aura, had a significantly higher proportion of non-ischemic penumbra than those without a history of migraine, and that the ischemic penumbra volume was smaller in those with aura, suggesting that patients with a history of migraine are more sensitive to cerebral ischemia.

BHI reduction reflects endothelial dysfunction and decreased cerebrovascular reserve, and is closely associated with cerebral small vessel damage. Song et al. (25) found that BHI was significantly reduced in cryptogenic stroke patients with RLS, and that BHI was negatively correlated with the location and severity of white matter hyperintensities. Lee et al. (21) demonstrated that low MCA-BHI was independently associated with the number of deep white matter hyperintensities in migraine patients, and that migraine modified the effect of MCA-BHI on deep white matter hyperintensities. Xie et al. (22) further confirmed in a larger sample (397 migraine patients) that migraine patients with RLS had significantly lower BHI than those without RLS, and that reduced BHI was an independent influencing factor for white matter hyperintensities. The clinical value of BHI is also related to the prognosis of cerebrovascular patients: Bian et al. (26) found that BHI was negatively correlated with disease severity in patients with symptomatic leukoaraiosis, and that patients with lower BHI had worse cognitive function; Troisi et al. (27) reported that BHI was positively correlated with activities of daily living in patients with non-lacunar stroke, and that patients with preserved BHI had better neurological recovery. These findings indicate that BHI reduction is not merely a physiological variation, but rather a functional marker closely associated with cerebral small vessel damage and post-stroke functional prognosis.

## 5 Conclusion

RLS associated with PFO is significantly associated with reduced cerebrovascular reactivity in migraine patients with aura, with the degree of reduction showing a dose-dependent relationship with RLS grade and shunt persistence, particularly in the posterior circulation. BHI reduction is associated with cerebral small vessel damage and poor functional prognosis, suggesting that RLS-related CVR impairment may have clinical significance. This study provides new data supporting the understanding of the impact of PFO-related RLS on CVR in migraine patients.

## 6 Limitations

This study has several limitations. First, the single-center cross-sectional design precludes the establishment of causal relationships. Second, the sample size was limited (*n* = 62), with only eight and 12 patients in the RLS grade 2 and grade 3 subgroups, respectively. Some subgroup comparisons had insufficient statistical power [e.g., PFO (–) vs. grade 2: power = 0.43], and negative results among subgroups should be interpreted with caution. Third, we did not perform multivariable adjustment for potential confounders or correction for multiple comparisons. Although baseline characteristics were balanced between groups, the possibility of residual confounding and type I error cannot be completely excluded. The main conclusions are based on overall ANOVA tests (which control the overall error rate) and their reliability is not affected; however, subgroup comparison results should be considered exploratory. Fourth, although BHI measurement was standardized, it still relies on patient cooperation and may introduce bias. Fifth, only migraine patients with aura were enrolled, and all were of Han Chinese ethnicity; therefore, the generalizability of our conclusions to patients with migraine without aura or to other ethnic populations requires caution.

Future studies should expand the sample size and adopt multicenter prospective designs to further validate these findings.

## Data Availability

The original contributions presented in the study are included in the article/supplementary material, further inquiries can be directed to the corresponding author/s.
